# Limits on reliable information flows through stochastic populations

**DOI:** 10.1371/journal.pcbi.1006195

**Published:** 2018-06-06

**Authors:** Lucas Boczkowski, Emanuele Natale, Ofer Feinerman, Amos Korman

**Affiliations:** 1 CNRS, IRIF, Université Paris Diderot, Paris, France; 2 Algorithms and Complexity Department, Max-Planck-Institut für Informatik, Saarbrücken, Germany; 3 Department of Physics of Complex Systems, Weizmann Institute of Science, Rehovot, Israel; University of California Irvine, UNITED STATES

## Abstract

Biological systems can share and collectively process information to yield emergent effects, despite inherent noise in communication. While man-made systems often employ intricate structural solutions to overcome noise, the structure of many biological systems is more amorphous. It is not well understood how communication noise may affect the computational repertoire of such groups. To approach this question we consider the basic collective task of rumor spreading, in which information from few knowledgeable sources must reliably flow into the rest of the population. We study the effect of communication noise on the ability of groups that lack stable structures to efficiently solve this task. We present an impossibility result which strongly restricts reliable rumor spreading in such groups. Namely, we prove that, in the presence of even moderate levels of noise that affect all facets of the communication, no scheme can significantly outperform the trivial one in which agents have to wait until directly interacting with the sources—a process which requires linear time in the population size. Our results imply that in order to achieve efficient rumor spread a system must exhibit either some degree of structural stability or, alternatively, some facet of the communication which is immune to noise. We then corroborate this claim by providing new analyses of experimental data regarding recruitment in *Cataglyphis niger* desert ants. Finally, in light of our theoretical results, we discuss strategies to overcome noise in other biological systems.

## Author summary

Biological systems must function despite inherent noise in their communication. Systems that enjoy structural stability, such as biological neural networks, could potentially overcome noise using simple redundancy-based procedures. However, when individuals have little control over who they interact with, it is unclear what conditions would prevent runaway error accumulation. This paper takes a general stance to investigate this problem, concentrating on the basic information-dissemination task of rumor spreading. Drawing on a theoretical model, we prove that fast rumor spreading can only be achieved if some part of the communication setting is either stable or reliable. We then provide empirical support for this claim by conducting new analyses of data from experiments on recruitment in desert ants.

## Introduction

Systems composed of tiny mobile components must function under conditions of unreliability. In particular, any sharing of information is inevitably subject to communication noise. The effects of communication noise in distributed living systems appears to be highly variable. While some systems disseminate information efficiently and reliably despite communication noise [1–5], others generally refrain from acquiring social information, consequently losing all its potential benefits [6–8]. It is not well understood which characteristics of a distributed system are crucial in facilitating noise reduction strategies and, conversely, in which systems such strategies are bound to fail. Progress in this direction may be valuable towards better understanding the constraints that govern the evolution of cooperative biological systems.

Computation under noise has been extensively studied in the computer science community. These studies suggest that different forms of error correction (*e.g.*, redundancy) are highly useful in maintaining reliability despite noise [9–12]. All these, however, require the ability to transfer significant amount of information over stable communication channels. Similar redundancy methods may seem biologically plausible in systems that enjoy stable structures, such as brain tissues.

The impact of noise in stochastic systems with ephemeral connectivity patterns is far less understood. To study these, we focus on *rumor spreading*—a fundamental information dissemination task that is a prerequisite to almost any distributed system [13–16]. The literature on *rumor spreading* is quite vast and encompasses different disciplines over the last decades [17, 18]. For a succinct overview as for theoretical computer science, see Section *Related works in computer science* in the Supplementary Information.

A successful and efficient rumor spreading process is one in which a large group manages to quickly learn information initially held by one or a few informed individuals. Fast information flow to the whole group dictates that messages be relayed between individuals. Similar to the game of Chinese Whispers, this may potentially result in runaway buildup of noise and loss of any initial information [19]. It currently remains unclear what are the precise conditions that enable fast rumor spreading. On the one hand, recent works indicate that in some models of random noisy interactions, a collective coordinated process can in fact achieve fast information spreading [20, 21]. These models, however, are based on *push* operations that inherently include a certain reliable component (see more details in Section *Separation between PUSH and PULL*). On the other hand, other works consider computation through noisy operations, and show that several distributed tasks require significant running time [22]. The tasks considered in these works (including the problem of learning the input bits of all processors, or computing the parity of all the inputs) were motivated by computer applications, and may be less relevant for biological contexts. Moreover, they appear to be more demanding than basic tasks, such as rumor spreading, and hence it is unclear how to relate bounds on the former problems to the latter ones.

In this paper we take a general stance to identify limitations under which reliable and fast rumor spreading cannot be achieved. Modeling a well-mixed population, we consider a passive communication scheme in which information flow occurs as one agent observes the cues displayed by another. If these interactions are perfectly reliable, the population could achieve extremely fast rumor spreading [16]. In contrast, here we focus on the situation in which messages are noisy. Informally, our main theoretical result states that fast rumor spreading through large populations can only be achieved if either

- the system exhibits some degree of structural stability, or
- some facet of the pairwise communication is immune to noise.

In fact, our lower bounds hold even when individuals are granted unlimited computational power and even when the system can take advantage of complete synchronization. In light of these theoretical results, we then turn to discuss several examples of information sharing in distributed biological systems. We provide new analyses of the efficiency of information dissemination during recruitment by desert ants. These suggest that this system lacks reliability in all its communication components, and its deficient performances qualitatively validate our predictions. Finally, we revisit existing rumor spreading solutions in large biological systems and discuss different strategies for confronting noise.

### The problem

An intuitive description of the model follows. For more precise definitions, see, Section *The models* in the Supplementary Information.

Consider a population of *n* *agents*. Thought of as computing entities, assume that each agent has a discrete internal *state*, and can execute randomized algorithms—by internally flipping coins. In addition, each agent has an *opinion*, which we assume for simplicity to be binary, *i.e.*, either 0 or 1. A small number, *s*, of agents play the role of *sources*. Source agents are aware of their role and share the same opinion, referred to as the *correct opinion*. The goal of all agents is to have their opinion coincide with the correct opinion.

To achieve this goal, each agent continuously displays one of several *messages* taken from some finite alphabet Σ. Agents interact according to a random pattern, termed as the *parallel-PULL* model: In each round $t\in N^{+}$, each agent *u* observes the message currently displayed by another agent *v*, chosen independently and uniformly at random from all agents. Importantly, communication is noisy, hence the message observed by *u* may differ from that displayed by *v*. More precisely, for any *m*, *m*′ ∈ Σ, let *P*_*m*,*m*′_ be the probability that, any time some agent *u* observes an agent *v* holding some message *m* ∈ Σ, *u* actually receives message *m*′. The probabilities *P*_*m*,*m*′_ define the entries of the noise-matrix *P* [21], which does not depend on time.

The noise is characterized by a *noise parameter* *δ* > 0. Our model encapsulates a large family of noise distributions, making our bounds highly general. Specifically, the noise distribution can take *any* form, as long as it satisfies the following criterion.

**Definition 1 (*δ*-uniform noise)** *We say that the noise is δ-uniform if P*_*m*,*m*′_ ≥ *δ for any m*, *m*′ ∈ Σ.

When messages are noiseless, it is easy to see that the number of rounds that are required to guarantee that all agents hold the correct opinion with high probability is O(logn) [16]. In what follows, we aim to show that when the *δ*-uniform noise criterion is satisfied, the number of rounds required until even one non-source agent can be moderately certain about the value of the correct opinion is very large. Specifically, thinking of *δ* and *s* as constants independent of the population size *n*, this number of rounds is at least Ω(*n*).

To prove the lower bound, we will bestow the agents with capabilities that far surpass those that are reasonable for biological entities. These include:

- Unique identities: Agents have unique identities in the range {1, 2, …*n*}. When observing agent *v*, its identity is received without noise.
- Complete knowledge of the system: Agents have access to all parameters of the system (including *n*, *s*, and *δ*) as well as to the full knowledge of the initial configuration except, of course, the correct opinion and the identity of the sources. In addition, agents have access to the results of random coin flips used internally by all other agents.
- Full synchronization: Agents know when the execution starts, and can count rounds.

We show that even given this extra computational power, fast convergence cannot be achieved. All the more so, fast convergence is impossible under more realistic assumptions.

## Results

The purpose of this work is to identify limitations under which efficient rumor spreading would be impossible. Our main result is theoretical and, informally, states that when all components of communication are noisy fast rumor spreading through large populations is not feasible. In other words, our results imply that fast rumor spreading can only be achieved if the system either exhibits some degree of structural stability or that some facet of its communication is immune to noise. These results in hand, a next concern is how far our highly theoretical analysis can go in explaining actual biological systems.

Theoretical results with a high degree of generality may hold relevance to a wider range of biological systems. Lower bound and impossibility results follow this approach. Indeed, impossibility results from physics and information theory have previously been used to further the understanding of several biological systems [23, 24]. The results we present here are, similarly, in the form of lower bounds but, this time, they are derived from the realm of distributed computation. As such, our theorems are general enough to constrain the performances of a vast class of computational systems regardless of their particulars or the specific computational algorithms which they apply. This generality stretches over to biology and can provide us with fundamental lessons regarding the limitations faced by distributed biological systems [24–26].

While the generality of our lower bound results makes them relevant to a large number of biological systems it also constitutes a weakness. Namely, the assumptions on which such theorems are based are not tailored to describe a particular system. This implies that comparisons between the model assumptions and the actual details of a specific system will not be perfect. Nevertheless, we show how our theoretical results can shed light on some non-trivial behaviors in a specific biological system whose characteristics are close enough to the underlying theoretical assumptions (see Section *Recruitment in desert ants*). Particularly, we empirically show that when desert ants communicate information regarding a new food source they are subject to limitations which are similar to those assumed by our model. We then demonstrate a non-trivial slowdown in the speed at which information spreads through the system as a function of group size. Despite the non-perfect matching between the theoretical assumption and the biological system, this non-trivial result stands in direct accordance with our theoretical lower bounds.

Distributed computing provides an effective means of studying biological groups [27–30]. However, to the best of our knowledge, there are no examples in which algorithmic lower bounds, one of distributed computing most powerful tools, have been applied to a particular living system. This work uses lower bounds to provide insights into non-trivial dynamics observed during ant recruitment behavior.

### Theoretical results

In all the statements that follow we consider the parallel-PULL model satisfying the *δ*-uniform noise criterion, with *cs*/*n* < *δ* ≤ 1/|Σ| for some sufficiently large constant *c*, where the upper bound follows from the criterion given in Definition 1. Hence, the previous lower bound on *δ* implies a restriction on the alphabet size, specifically, |Σ| < *n*/(*cs*).

**Theorem 1.1** *Any rumor spreading protocol cannot converge in less than* $\Omega (\frac{n\delta }{s^{2}{\left(1-\delta \left|\Sigma \right|\right)}^{2}})$ *rounds*.

Observe that the lower bound we present loses relevance when *s* is of order greater than $\sqrt{n}$, as our proof technique becomes uninformative in presence of a large number of sources (see *Remark 2* in the Supplementary Information). Recall also that we assume that a source is aware that it is a source, but if it wishes to identify itself as such to agents that observe it, it must encode this information in a message, which is, in turn, subject to noise. We also consider the case in which an agent can reliably identify a source when it observes one (that is, this information is not noisy). For this case, the following lower bound, which is weaker than the previous one but still polynomial, apply (see also the S1 Text, Detectable sources):

**Corollary 1.1** *Assume that sources are reliably detectable. There is no rumor spreading protocol that converges in less than* $\Omega ({\left(\frac{n\delta }{s^{2}{\left(1-\delta \left|\Sigma \right|\right)}^{2}}\right)}^{1/3})$ *rounds*.

**Fig 1:**
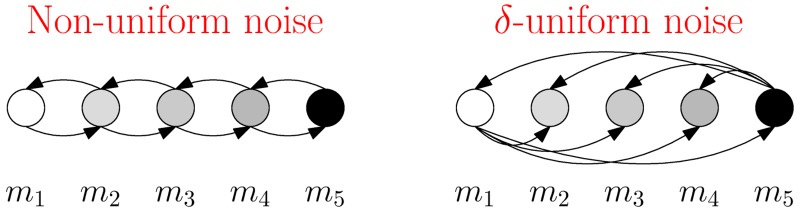
Non-uniform noise vs. uniform noise. On the left, we consider an example with non-uniform noise. Assume that the message vocabulary consists of 5 symbols, that is, Σ = {*m*_1_, *m*_2_, *m*_3_, *m*_4_, *m*_5_}, where *m*_1_ = 0 and *m*_5_ = 1, represent the opinions. Assume that noise can occur only between consecutive messages. For example, *m*_2_ can be observed as either *m*_2_, *m*_3_ or *m*_1_, all with positive constant probability, but can never be viewed as *m*_4_ or *m*_5_. In this scenario, the population can quickly converge on the correct opinion by executing the following. The sources always display the correct opinion, *i.e.*, either *m*_1_ or *m*_5_, and each other agent displays *m*_3_ unless it has seen either *m*_1_ or *m*_5_ in which case it adopts the opinion it saw and displays it. In other words, *m*_3_ serves as a default message for non-source agents, and *m*_1_ and *m*_5_ serve as attracting sinks. It is easy to see that the correct opinion will propagate quickly through the system without disturbance, and within O(logn) number of rounds, where *n* is the size of the population, all agents will hold it with high probability. In contrast, in the case of *δ*-uniform noise as depicted on the right picture, if every message can be observed as any other message with some constant positive probability (for clarity, some of the arrows have been omitted from the sketch), then convergence cannot be achieved in less than Ω(*n*) rounds, as Theorem 1.1 dictates.

Our results suggest that, in contrast to systems that enjoy stable connectivity, structureless systems are highly sensitive to communication noise (see Fig 1). More concretely, the two crucial assumptions that make our lower bounds applicable are: 1) stochastic interactions, and 2) *δ*-uniform noise (Fig 1, right hand panel). When agents can stabilize their interactions the first assumption is violated. In such cases, agents can overcome noise by employing simple error-correction techniques, *e.g.*, using redundant messaging or waiting for acknowledgment before proceeding to the next action. As demonstrated in Fig 1, (left hand panel), when the noise is not uniform, it might be possible to overcome it with simple techniques based on using default neutral messages, and employing exceptional distinguishable signals only when necessary.

### Recruitment in desert ants

Our theoretical results assert that efficient rumor spreading in large groups could not be achieved without some degree of communication reliability. An example of a biological system whose communication reliability appears to be deficient in all of its components is recruitment in *Cataglyphis niger* desert ants. In this species, when a forager locates an oversized food item, she returns to the nest to recruit other ants to help in its retrieval [31, 32].

**Fig 2:**
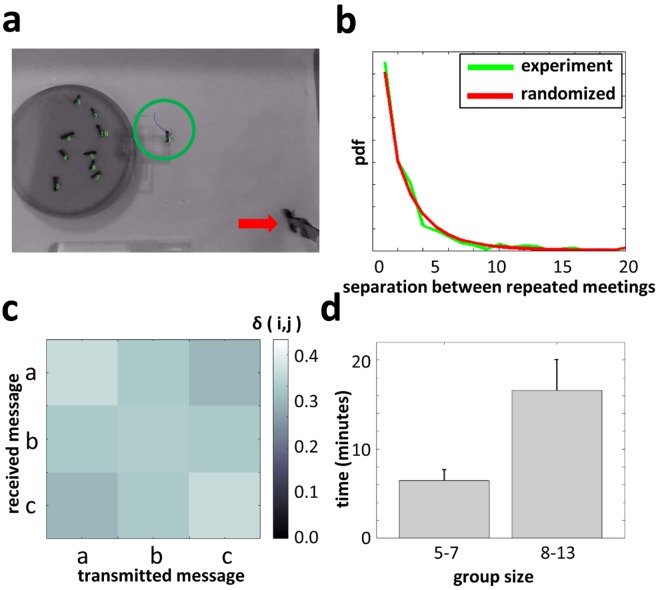
Unreliable communication and slow recruitment by desert ant (*Cataglyphis niger*). **a.** The experimental setup. The recruiter ant (circled) returns to the nest’s entrance chamber (dark, 9cm diameter, disc) after finding the immobilized food item (arrow). Group size is ten. **b.** A *pdf* of the number of interactions that an ant experiences before meeting the same ant twice. The *pdf* is compared to uniform randomized interaction pattern. Data summarizes *N* = 671 interactions from seven experiments with a group size of 6 ants. **c.** Interactions of stationary ants with moving ants were classified into three different messages (‘a’ to ‘c’) depending on the moving ants’ speed. The noise at which messages were confused with each other was estimated according to the response of the recipient, initially stationary, ants (see Materials and methods). Gray scale indicates the estimated overlap between every two messages *δ*(*i*, *j*). Note *δ* = min(*δ*(*i*, *j*)) ≈ 0.3. Data collected over *N* = 278 interactions. **d.** The mean time it takes an ant that is informed about the food to recruit two nest-mates to exit the nest is presented for two group size ranges. Error bars represent standard error of the means over *N* = 24 experiments.

In our experimental setup, summarized in Fig 2, recruitment occurs in the small area of the nest’s entrance chamber (Fig 2a). We find that within this confined area, the interactions between ants follow a near uniformly random meeting pattern [33]. In other words, ants seem to have no control over which of their nest mates they will meet next (Fig 2b). This random meeting pattern approximates the first main assumption of our model. Another of the model’s assumptions is that ants interact in parallel. This implies that the interaction rate per ant be constant and independent of group size. Indeed, the empirical rate of interaction during the recruitment process was measured to be 0.82 ± 0.07 (mean ± sem, *N* = 44) interactions per minute per ant and induces a small increase with group size: 0.62 ± 0.13 for two ants (*N* = 8) and 1 ± 0.2 for a group sizes of 9-10 (*N* = 5).

It has been shown that recruitment in *Cataglyphis niger* ants relies on rudimentary alerting interactions [34, 35] which are subject to high levels of noise [32]. Moreover, the information an ant passes in an interaction can be attributed solely to her speed before the interaction [32]. Binning ant speeds into three discrete messages and measuring the responses of stationary ants to these messages, we can estimate the probabilities of one message to be mistakenly perceived as another one (see Estimating *δ* in the Methods). We find that this communication is extremely noisy which complies with the uniform-noise assumption with a *δ* of approximately 0.3 (Fig 2c). While artificially dividing the continuous speed signals into a large number of discrete messages (thus creating a larger alphabet) would inevitably decrease *δ*, this is not supported by our empirical data (see Section Methods).

Finally, the interaction scheme, as exhibited by the ants, can be viewed somewhere in-between the noisy-push and the noisy-pull models. Moving ants tend to initiate more interaction [32] and this may resemble, at first glance, a noisy-push interaction scheme. However, the ants’ interactions actually share characteristics with noisy-pull communication. Mainly, ants cannot reliably distinguish an ant that attempts to transmit information from any other non-communicating individual [32]. The fact that a receiver ant cannot be certain that a message was indeed communicated to her coincides with the lack of reliability in information transmission in line with our theoretical assumptions (see more details on this point in the Section *Separation between PUSH and PULL*).

Given the coincidence between the communication patterns in this ant system and the requirements of our lower bound we expect long delays before any uninformed ant can be relatively certain that a recruitment process is occurring. We therefore measured the time it takes an ant, that has been at the food source, to recruit the help of two nest-mates for different total group size. One might have expected this time to be independent of the group size or even to decrease as two ants constitute a smaller fraction of larger groups. To the contrary, we find that the time until the second ant is recruited increases with group size (*p* < 0.05 Kolmogorov-Smirnov test over *N* = 24 experiments, see Fig 2d).

Our theoretical results set a lower bound on the minimal time it takes uninformed ants to be recruited. Note that our lower bounds actually correspond to the time until *any* individual can be sure with more than 2/3 probability of the rumor. In the context of the ant recruitment experiment this means that if an ant goes out of the nest only if she is sure with some probability that there is a reason to exit, then the lower bounds correspond to the time until the first, and similarly the second (see Fig 2d), ants exit the nest.

Our lower bound is linear in the group size (Theorem 1.1). Note that this does not imply that the ants’ biological algorithm matches the lower bound and must be linear as well. Rather, our theoretical results qualitatively predict that as group size grows, recruitment times must eventually grow as well. This stands in agreement with Fig 2d. Thus, in this system, inherently noisy interactions on the microscopic level have direct implications on group level performance.

### Overview of the main lower bound proof

Here, we provide the intuition for our main theoretical result, Theorem 1.1. For a formal proof please refer to the S1 Text, The lower bounds. The proof can be broken into three parts and, below, we refer to each of them separately.

#### Part I. From parallel-PULL to broadcast-PULL

Consider an efficient protocol P for the parallel-PULL setting. The first part of the proof shows how P can be used to produce a protocol $P^{\prime }$ that operates in another model, called *broadcast-PULL*. In this latter model, at each time step $t\in N^{+}$ one agent is chosen u.a.r. and all agents observe it, receiving the same noisy sample of its message. The running time of the resulting protocol $P^{\prime }$ will be *n* times the running time of P. The construction of $P^{\prime }$ builds on the permissive assumptions we employ regarding the power of computation of agents and their unique identities in {1, 2, …*n*}. In $P^{\prime }$, agents divide time steps in the broadcast-PULL model into *rounds*, each composed of precisely *n* time steps. For an integer *i*, where 1 ≤ *i* ≤ *n*, during the *i*-th step of each round, all agents receive an observation, but *n* − 1 of them ignore it. Specifically, only agent (*i* mod *n*)+1 keeps the observation. The agent will then wait until the end of the round to actually process this observation according to P. This ensures that when a round is completed, each agent processes precisely one independent uniform sample from the configuration of the previous round, as it would in a round of the parallel-PULL model. This draws a precise injection from rounds in broadcast-PULL and rounds in parallel-PULL. This construction implies that to prove Theorem 1.1 it is enough to prove that there is no rumor spreading protocol in the broadcast-PULL model that converges in less than $\Omega (\frac{n^{2}\delta }{s^{2}{\left(1-\delta \left|\Sigma \right|\right)}^{2}})$ rounds.

#### Part II. From broadcast-PULL to a statistical inference problem

To establish the desired lower bound, we next show how the rumor spreading problem in the broadcast-PULL model relates to a statistical inference test. That is, from the perspective of a given agent, the rumor spreading problem can be understood as the following: Based on a sequence of noisy observations, the agent should be able to tell whether the correct opinion is 0 or 1. We formulate this problem as a specific task of distinguishing between two random processes, one originated by running the protocol assuming the correct opinion is 0 and the other assuming it is 1.

**Fig 3:**
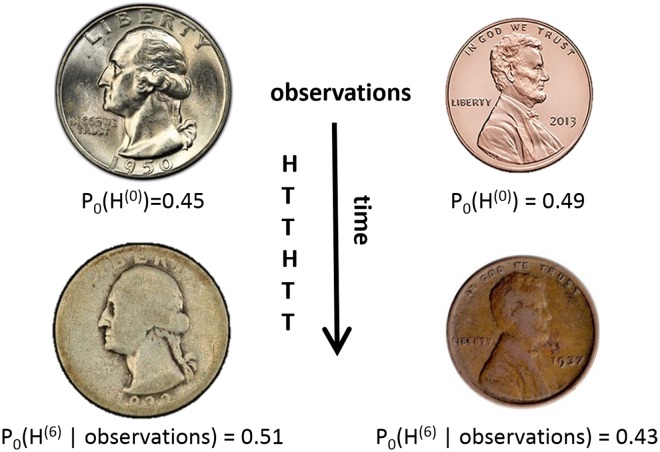
Distinguishing between two types of coins. On the top there are two possible coins with slightly different distributions for yielding a head (*H*) or a tail (*T*). (We depicted two possible outcomes but our model can account for more.) Given a sequence of observations (corresponding to the random outcomes of coin tosses), the goal of the observer is to guess the coin type being used (either 0 or 1). The wear induced by tossing the coins may, with time, change the probability that they land on either heads or tails in a way that depends on the coin type as well as on the previous toss outcomes (observations). In particular, notice that without a change in the probability of heads in our example we would not obtain a posterior probability 0.51 starting with a prior of 0.45 after six coin flips involving two heads. *P*_*j*_(*H*^(*t*)^ ∣ observations) for *j* ∈ {0, 1} denotes the probability of Coin of type *j* to yield *H* given the particular sequence of *t* observations. Here, this sequence is *H*, *T*, *T*, *H*, *T*, *T*. At the beginning of the next round, *i.e.*, the 7th round, |*ε*^(*t* = 6)^|_1_ measures how “far” the the *H* vs *T* distribution generated by the worn down coin 1 is from the same distribution as generated by the worn down coin 2. More precisely, for the case above, |*ε*^(6)^|_1_ = |*P*_0_(*H* ∣ observations) − *P*_1_(*H* ∣ observations)| + |*P*_0_(*T* ∣ observations) − *P*_1_(*T* ∣ observations)| = |0.51 − 0.43| + |0.49 − 0.57| = 0.16. The parameter *ε* bounds all possible |*ε*^(*t*)^|_1_ from above.

One of the main difficulties lies in the fact that these processes may have a memory. At different time steps, they do not necessarily consist of independent draws of a given random variable. In other words, the probability distribution of an observation not only depends on the correct opinion, on the initial configuration and on the underlying randomness used by agents, but also on the previous noisy observation samples and (consequently) on the messages agents themselves choose to display on that round. An intuitive version of this problem is the task of distinguishing between two (multi-valued) biased coins, whose bias changes according to the previous outcomes of tossing them (*e.g.*, due to wear). See Fig 3 for an illustration.

Despite this apparent complexity, we show that the difficulty of this distinguishing task can be captured by two scalar parameters, denoted *ε* and *δ*. Parameter *δ* lower bounds the probability for any observation to be attained (given any sequence of observations). Parameter *ε* captures the extent to which the processes are ‘similar’. More specifically, at round *t*, given a sequence of previous observations, denoted *x*^(<*t*)^, the next observation has the same probability to be attained in each process, up to an *ε* additive term (see Fig 3). A crucial observation is that *ε* is very small, precisely, *ε* = Θ(*s*(1 − *δ*|Σ|)/*n*). This follows from the fact that given *x*^(<*t*)^, the behavior of non-source agents in the two processes is the same, regardless of the value of the correct opinion. Indeed, internally, an agent is only affected by its initial knowledge, the randomness it uses, and the sequence of observations it sees. This means that at round *t*, the processes would differ only if the agent to be observed on that round happens to be a source (which happens with probability *s*/*n*) and, on top of that, the observed message is not the changed by noise (which accounts for the factor (1 − *δ*|Σ|)). However, a small value of *ε* is not enough to ensure slow running time. Indeed, even though the *t*’th observation may be distributed almost the same, if it happens that some observation can be attained only in one process, then seeing such an observation would immediately allow the observer to distinguish the two processes. A sufficiently large *δ* prevents the aforementioned scenario.

#### Part III. A lower bound for the statistical test problem

The last step of the proof shows that at least Ω(*δ*/*ε*^2^) samples are required in order to solve any distinguishing task with parameters *δ* and *ε*. The proof involves notions from Statistical Hypothesis Testing such as the Kullback-Leibler (KL) divergence (see, *e.g.*, Chapter 5 in [36]). For example, generalizing known results, we show that, if $P_{0}^{\left(\leq t\right)}$ and $P_{1}^{\left(\leq t\right)}$ are the two distributions of observations up to time *t*, any distinguishing algorithm must satisfy that the error probability is at least $1-\sqrt{KL(P_{0}^{\left(\leq t\right)},P_{1}^{\left(\leq t\right)})}$ (see Rumor Spreading and hypothesis testing, S1 Text). Hence, for the probability of error to be small, the term $KL(P_{0}^{\left(\leq t\right)},P_{1}^{\left(\leq t\right)})$ must be large. To calculate the KL-divergence one often uses a tensorization lemma, but this could not be used in our case since the observations in different rounds are not independent. Instead, we use the more general Chain Rule identity (see [36]). This allows us to focus on the KL-divergence of every round separately rather than of the whole sequence. In contrast to the fully independent case, we also condition on the previous draws, on the randomness used by agents, and on the initial configuration. Finally, we obtain: $KL\left(P_{0}^{\left(\leq t\right)},P_{1}^{\left(\leq t\right)}\right)=O\left(\frac{t\varepsilon^{2}}{\delta }\right)$. This implies that the number of observations *t* needs to be of order *δ*/*ε*^2^ to make the error less than, say 1/3. This bound translates to a lower bound of Ω(*n*^2^*δ*/(*s*^2^(1 − *δ*|Σ|)^2^)) steps for the broadcast-PULL model and hence a lower bound of Ω(*nδ*/(*s*^2^(1 − *δ*|Σ|)^2^)) rounds for the parallel-PULL model.

### Generalizations

Several of the assumptions discussed earlier for the parallel-PULL model were made for the sake of simplicity of presentation. In fact, our results can be shown to hold under more general conditions, that include: 1) different rate for sampling a source, and 2) a more relaxed noise criterion. In addition, our theorems were stated with respect to the parallel-PULL model. In this model, at every round, each agent samples a single agent u.a.r. In fact, for any integer *k*, our analysis can be applied to the model in which, at every round, each agent observes *k* agents chosen u.a.r. In this case, the lower bound would simply reduce by a factor of *k*. Our analysis can also apply to a sequential variant, in which in each time step, two agents *u* and *v* are chosen u.a.r from the population and *u* observes *v*. In this case, our lower bounds would multiply by a factor of *n*, yielding, for example, a lower bound of Ω(*n*^2^) in the case where *δ* and *s* are constants. Observe that the latter increase is not surprising as each round in the parallel-PULL model consists of *n* observations, while the sequential model consists of only one observation in each time step. See more details in the Supplementary Information.

## Discussion

### Exponential separation between PUSH and PULL

Our lower bounds on the parallel-PULL model (where agents observe other agents) should be contrasted with known results in the parallel-PUSH model (this is the push equivalent to parallel-PULL model, where in each round each agent may or may not actively push a message to another agent chosen u.a.r.). Although never proved, and although their combination is known to achieve more power than each of them separately [16], researchers often view the parallel-PULL and parallel-PUSH models as very similar on complete communication topologies. Our lower bound result, however, undermines this belief, proving that in the context of noisy communication, there is an exponential separation between the two models. Indeed, when the noise level is constant for instance, convergence (and in fact, a much stronger convergence than we consider here) can be achieved in the parallel-PUSH using only logarithmic number of rounds [20, 21], by a simple strategy composed of two stages. The first stage consists of providing all agents with a guess about the source’s opinion, in such a way that ensures a non-negligible bias toward the correct guess. The second stage then boosts this bias by progressively amplifying it. A crucial aspect in the first stage is that agents remain silent until a certain point in time that they start sending out messages. This prevents agents from starting to spread information before they have sufficiently reliable knowledge and allows for a balanced control of the rumor spread. More specifically, marking an edge corresponding to a message received for the first time by an agent, the set of marked edges forms a spanning tree of low depth, rooted at the source. The depth of such tree can be interpreted as the deterioration of the message’s reliability. On the other hand, as shown here, in the parallel-PULL model, even with the synchronization assumption, rumor spreading cannot be achieved in less than a linear number of rounds.

Perhaps the main reason why these two models are often considered similar is that with an extra bit in the message, a PUSH protocol can be *approximated* in the PULL model, by letting this bit indicate whether the agent in the PUSH model was aiming to push its message. However, for such a strategy to work, this extra bit has to be reliable. Yet, in the noisy PULL model, no bit is safe from noise, and hence, as we show, such an approximation cannot work. In this sense, the extra power that the noisy PUSH model gains over the noisy PULL model, is that the very fact that one node attempts to communicate with another is reliable. This, seemingly minor, difference carries significant consequences.

### Strategies to overcome noise in biological systems

Communication in man-made computer networks is often based on reliable signals which are typically transferred over highly defined structures. These allow for ultra-fast and highly reliable calculations. Biological networks are very different from this and often lack reliable messaging, well defined connectivity patterns or both. Our theoretical results seem to suggest that, under such circumstances, efficient spread of information would not be possible. Nevertheless, many biological groups disseminate and share information, and, often, do so reliably. Next, we discuss information sharing in biological systems within the general framework of our lower-bounds.

The correctness of the lower bounds relies on two major assumptions: 1) stochastic interactions, and 2) uniform noise. Communication during desert ant recruitment complies with both these assumptions (see Fig 2b and 2c) and indeed the speed at which messages travel through the group (see Fig 2d) is low. Below, we discuss several biological examples where efficient rumor spreading is achieved. We expect that, in these examples, at least one of the assumptions mentioned above should break adding some degree of reliability to the overall communication. The group can then utilize this reliability and follow one of the strategies mentioned in Section *Theoretical results*, in order to yield reliable collective performance. We begin by discussing examples that violate the first assumption, namely, that of stochastic interactions, and then discuss examples that violate the second assumption, namely, uniform noise.

#### Stable connectivity as a means to overcome noise

Synaptic connectivity in the mammalian brain is known to be highly noisy [37]. However, this cellular-level noise has little effect on the global function of brains which are highly reliable. A major factor that allows this restoration of reliability are the structural stability and redundancies that characterize brain connectivity. It is well understood how such properties can allow for fast and efficient propagation of electrical signals through large neuronal populations [1, 2].

Animal groups can also benefit from stable connectivity to enhance the reliability of rumor spreading. An example comes from house-hunting rock ants [38]. When these ants commence their move between nest sites, the information regarding the location of the target nest is held by only a few scout ants, that then disseminate it to the entire colony. To communicate this information, the ants engage in prolonged interactions which include the formation of stable pairs that walk in tandem towards the target nest and frequently contact each other [39]. These tandem run interactions allow for highly reliable communication and permit the follower ant to lead a subsequent interaction such that the rumor can continue to spread efficiently. While this redundancy of multiple interaction allows for the efficient flow of information, it comes at the inevitable price of long interaction durations [40].

#### Non-uniform noise

When the physical structure of a group is not well defined, the importance of reliable messaging schemes grows. In flocks of birds and schools of fish, changes in the behavior of a single individual can be relayed across a series of local interactions [41], and generate a response wave that travels across the entire group [3–5]. It has been shown that information can travel faster in flocks that display a higher level of alignment [42]. Within the context of our analysis, as a group becomes more ordered it becomes easier to distinguish a sudden directional change by an informed individual from the random velocity fluctuations of uninformed birds. Besides a possibly reduced level of uniformity in the interactions implied by the additional spatial structure, the reliability of information transfer violates the *δ*-uniform noise assumption and allows for fast and reliable directional changes on the collective scale.

As noted above, push-type communication is another route which may potentially add sufficient reliability to support rumor spreading. In this sense, what distinguishes push from pull is the trait by which a non-message cannot be confused with a message. An example for the usefulness of an active push behavior comes from alarm behavior in ants. A single ant sensing danger can actively excrete discrete volatile alarm pheromones that are sensed by a large number of group members and elicit panic or attack responses [43, 44]. Conversely, no ant would secrete these distinct pheromones unless she directly perceived danger or sensed the alarm signal. Therefore, when an ant senses an alarm pheromone the only possibility is that one of her nest mates has sensed danger. As indicated by our theoretical lower bound, if alarm messages would be confused with non-alarming messages then such fast and reliable information spread would not be possible.

The difficulty of spreading information fast, as indicated by our theoretical results, is further consistent with the fact that, even in fully-cooperative groups, such as ants or bees, an animal that receives information from a conspecific will often not transfer it further before obtaining its own independent first-hand knowledge [40, 45–48].

Finally, we note that given the aforementioned discussion, our insight regarding the difficulty of functioning under uniform noise can serve an evolutionary explanation for the emergence of new communication signals (*e.g.*, alarming signal) that would be distinct from other signals, and prevent confusion.

## Methods

All experimental results presented in this manuscript are re-analyses of data obtained in *Cataglyphis niger* recruitment experiments [32]. In short, ants in the entrance chamber of an artificial nest were given access to a tethered food item just outside the nest’s entrance (Fig 2a). The inability of the ants to retrieve the food induced a recruitment process [32].

The reaction of the ants to this manipulation was filmed and the locations, speeds and interactions of all participating ants were extracted from the resulting videos.

### Calculation of *δ*

To estimate the noise parameter *δ* we used interactions between ants moving at three different speed ranges (measured in *cm*/*sec*), namely, ‘a’: 1-10, ‘b’: 10-20, and ‘c’: over 20 and “receiver” ants. Only interactions in which the receiver ant was initially stationary were used as to ensure that the state of these ants before the interaction is as similar as possible. The message alphabet is then assumed to be Σ = {*a*, *b*, *c*}. The response of a stationary ant *v* to the interaction was quantified in terms of her speed after the interaction.

An alphabet of three messages was used since the average responses of *v* to any two messages were significantly different (all *p*-values smaller than 0.01) justifying the fact that these are not artificial divisions of a continuous speed signal into a large number of overlapping messages. On the other hand, dividing the bins further (say, each bin divided into 2 equal bins) yielded statistically indistinguishable responses from the receiver (all p-values larger than 0.11). Therefore, our current data best supports a three letter alphabet.

Assuming equal priors to all messages in Σ, and given specific speed of the receiver ant, *v*, the probability that it was the result of a specific message *i* ∈ Σ was calculated as *p*_*i*_(*v*) = *p*(*v* ∣ *i*)/∑_*k*∈Σ_ *p*(*v* ∣ *k*), where *p*(*v* ∣ *j*) is the probability of responding in speed *v* after “observing” *j*. The probability *δ*(*i*, *j*) that message *i* was perceived as message *j* was then estimated as the weighted sum over the entire probability distribution measured as a response to *j*: *δ*(*i*, *j*) = ∑_*v*_ *p*(*v* ∣ *j*) ⋅ *p*_*i*_(*v*). The parameter *δ* can then be calculated using *δ* = min{*δ*(*i*, *j*) ∣ *i*, *j* ∈ Σ}.

## Supporting information

S1 Text Supporting information. (TEX)

S1 Table Data associated to Fig 2. (XLSX)

S2 Table Data associated to Fig 2. (XLSX)

S3 Table Data associated to Fig 2. (XLSX)
