# Correction: Limits on reliable information flows through stochastic populations

**DOI:** 10.1371/journal.pcbi.1006404

**Published:** 2018-08-09

**Authors:** The PLOS Computational Biology Staff

Supporting Information file S1 Text was published in the wrong format. The publisher apologizes for the error. The correct Supporting Information file is available below.

## Supporting information

S1 Text Supporting information. (PDF)
