# Supplementary material for: Correction: Limits on reliable information flows through stochastic populations
Source: PLoS Comput Biol. 2018 Aug 9;14(8):e1006404. doi: 10.1371/journal.pcbi.1006404 (PMC6084812; doi:10.1371/journal.pcbi.1006404)
Supplement: S1 Text — (PDF) [file pcbi.1006404.s001.pdf]

(Supplementary Information)
Limits on Reliable Information Flows through Stochastic Populations

Lucas Boczkowski Emanuele Natale Ofer Feinerman Amos Korman

The models

We consider a population of n agents that interact stochastically and aim to converge on a particular opinion held by few knowledgable individuals. For simplicity, we assume that the set of opinions contain two opinions only, namely, 0 and 1.

As detailed in this section, we shall assume that agents have access to significant amount of resources, often exceeding more reasonable and realistic assumptions. Since we are concerned with lower bounds, we do not lose generality from such permissive assumptions. These liberal assumptions will actually simplify our proofs. One of these assumptions is the assumption that each agent is equipped with a unique identity $id(v)$ in the range $\{1, 2, \ldots, n\}$ (see more details in Section *Liberal assumptions*).

Initial configuration

The initial configuration is described in several layers. First, the *neutral initial configuration* corresponds to the initial states of the agents, before the sources and the desired opinion to converge to are set. (The term neutral is motivated by a physical analogy, as opposed to a charged initial configuration.)

A random initialization is then applied to the given neutral initial configuration, which determines the set of sources and the opinion that agents need to converge to. This will result in what we call the *charged initial configuration.* It can represent, for example, an external event that was identified by few agents which now need to deliver their knowledge to the rest of the population.

Neutral initial configuration $\mathbf{x}^{(0)}$

Each agent v starts the execution with an *input* that contains, in addition to its identity:

- an initial *state* taken from some discrete set of states, and
- a binary *opinion* variable $\lambda_v \in \{0, 1\}$.

(The opinion of an agent could have been considered as part of the state of the agent. We separate these two notions merely for the presentation purposes.) The *neutral initial configuration* $\mathbf{x}^{(0)}$ is the vector whose i'th index, $\mathbf{x}_i^{(0)}$ for $i \in \{1, 2, \ldots, n\}$, is the input of the agent with identity i.

Charged initial configuration and correct opinion

The charged initial configuration is determined in three stages. The first corresponds to the random selection of sources, the second to the selection of the correct opinion, and the third to a possible update of states of sources, as a result of being selected as sources with a particular opinion.

1st stage - Random selection of sources. Given an integer $s \leq n$, a set S of size s is chosen uniformly at random (u.a.r) among the agents. The agents in S are called *sources.* Note that any agent has equal probability of being a source. We assume that each source knows it is a source, and conversely, each non-source knows it is not a source.

2nd stage - Random selection of correct opinion. In the main model we consider, after sources have been determined in the first stage, the sources are randomly initialized with an opinion, called the *correct opinion.* That is, a fair coin is flipped to determine an opinion in $\{0, 1\}$ and all sources are assigned with this opinion.

3rd stage - Update of initial states of sources. To capture a change in behavior as a result of being selected as a source with a particular opinion, we assume that once the opinion of a source u has been determined, the initial state of u may change according to some distribution $f_{source-state}$ that depends on (1) its identity, (2) its opinion, and (3) the neutral configuration. Each source samples its new state independently.

Alphabet and messages

Agents communicate by observing each other according to some random pattern (for details see the Section *Random interaction patterns*). To improve communication agents may choose which content, called *message*, they wish to reveal to other agents that observe them. Importantly, however, such messages are subject to noise.

More specifically, at any given time, each agent v (including sources) displays a message $m \in \Sigma$, where Σ is some finite alphabet. The alphabet Σ agents use to communicate may be richer than the actual information content they seek to disseminate, namely, their opinions. This, for instance, gives them the possibility to express several levels of certainty [15]. We can safely assume that the size of Σ is at least two, and that Σ includes both symbols 0 and 1. We are mostly concerned with the case where Σ is of constant size (*i.e.,* independent of the number of agents), but note that our results hold for any size of the alphabet Σ, as long as the noise criterion is satisfied (see below).

δ-uniform noise

When an agent u *observes* some agent v, it receives a sample of the message currently held by v. The noise in the sample is characterized by a *noise parameter* $0 < \delta \leq 1/2$. One of the important aspects in our theorems is that they are general enough to hold assuming *any* distribution governing the noise, as long as it satisfies the following noise criterion.

Definition 1 (The noise criterion with parameter δ)**.** *Any time some agent u observes an agent v holding some message $m \in \Sigma$, the probability that u actually receives a message m' is at least δ, for any $m' \in \Sigma$. We assume that all noisy samples are independent.*

Observe that the aforementioned criterion implies that $\delta \leq 1/|\Sigma|$, and that the case $\delta = 1/|\Sigma|$ corresponds to messages being completely random, and the rumor spreading problem is thus unsolvable.

We next define a weaker criterion, that is particularly meaningful in cases in which sources are more restricted in their message repertoire than general agents. This may be the case, for example, if sources always choose to display their opinion as their message (possibly together with some extra symbol indicating that they are sources). Formally, we define $\Sigma' \subseteq \Sigma$ as the set of possible messages that a source can hold together with the set of messages that can be observed when viewing a source (*i.e.,* after noise is applied). Our theorems actually apply to the following criterion, that requires that only messages in Σ' are attained due to noise with some sufficient probability.

Definition 2 (Σ'-relaxed noise ellipticity parameter δ)**.** *We say that the noise has Σ'-relaxed ellipticity δ if $P_{m,m'} \geq \delta$ for any $m \in \Sigma$ and $m' \in \Sigma'$.*

Random interaction patterns

We consider several basic interaction patterns. Our main model is the *parallel-$\mathcal{PULL}$* model. In this model, time is divided into *rounds*, where at each round $i \in \mathbb{N}^+$, each agent u independently selects an agent v (possibly $u = v$) u.a.r from the population and then u observes the message held by v. The *parallel-$\mathcal{PULL}$* model should be contrasted with the *parallel-$\mathcal{PUSH}$* model, in which u can choose between *sending* a message to the selected node v or doing nothing. We shall also consider the following variants of $\mathcal{PULL}$ model.

- *parallel-$\mathcal{PULL}$*(k). Generalizing *parallel-$\mathcal{PULL}$* for an integer $1 \leq k \leq n$, the *parallel-$\mathcal{PULL}$*(k) model allows agents to observe k other agents in each round. That is, at each round $i \in \mathbb{N}^+$, each agent independently selects a set of k agents (possibly including itself) u.a.r from the population and observes each of them.

- *sequential-$\mathcal{PULL}$*. In each time step $t \in \mathbb{N}^+$, two agents u and v are selected uniformly at random (u.a.r) among the population, and agent u observes v.

- *broadcast-$\mathcal{PULL}$*. At each time step $t \in \mathbb{N}^+$ one agent is chosen u.a.r. from the population and all agents observe it, receiving the same noisy sample of its message.

The *broadcast-$\mathcal{PULL}$* model is mainly used for technical considerations. We use it in our proofs as it simplifies our arguments while not harming their generality. Nevertheless, this broadcast model can also capture some situations in which agents can be seen simultaneously by many other agents, where the fact that all agents observe the same sample can be viewed as noise being originated by the observed agent.

Regarding the difference in time units between the models, since interactions occur in parallel in the *parallel-$\mathcal{PULL}$* model, one round in that model should informally be thought of as roughly n time steps in the *sequential-$\mathcal{PULL}$* or *broadcast-$\mathcal{PULL}$* model.

Liberal assumptions

As mentioned, we shall assume that agents have abilities that surpass their realistic ones. This does not only increases the generality of our lower bounds, but also simplifies their proofs. Specifically, the following liberal assumptions are considered.

- **Unique identities.** Each agent is equipped with a unique identity $id(v) \in \{1, 2, \ldots, n\}$, that is, for every two agents u and v, we have $id(u) \neq id(v)$. Moreover, whenever an agent u observes some agent v, we assume that u can infer the identity of v. In other words, we provide agents with the ability to reliably distinguish between different agents at no cost.

- **Unlimited internal computational power.** We allow agents to have unlimited computational abilities including infinite memory capacity. Therefore, agents can potentially perform arbitrarily complex computations based on their knowledge (and their id).

- **Complete knowledge of the system.** Informally, we assume that agents have access to the complete description of the system except for who are the sources and what is their opinion. More formally, we assume that each agent has access to:
  - the neutral initial configuration $\mathbf{x}^{(0)}$,
  - all the systems parameters, including the number of agents n, the noise parameter δ, the number of sources s, and the distribution $f_{source-state}$ governing the update the states of sources in the third stage of the charged initial configuration.

- **Full synchronization.** We assume that all agents are equipped with clocks that can count time steps (in *sequential-$\mathcal{PULL}$* or *broadcast-$\mathcal{PULL}$*) or rounds (in *parallel-$\mathcal{PULL}$*(k)). The clocks are synchronized, ticking at the same pace, and initialized to 0 at the beginning of the execution. This means, in particular, that if they wish, the agents can actually share a notion of time that is incremented at each time step.

- **Shared randomness.** We assume that algorithms can be randomized. That is, to determine the next action, agents can internally toss coins and base their decision on the outcome of these coin tosses. Being liberal, we shall assume that randomness is shared in the following sense. At the outset, an arbitrarily long sequence r of random bits is generated and the very same sequence r is written in each agent's memory before the protocol execution starts. Each agent can then deterministically choose (depending on its state) which random bits in r to use as the outcome of its own random bits. In particular, since agents are allowed to have distinct initial states (e.g. by having *unique identity labels*), they can choose to make use of disjoint sets of random bits, thus making use of independent random variables. On the other hand, the shared randomness also implies that, for example, two agents can possibly make use of the very same random bits or merely observe the outcome of the random bits used by the other agents. Furthermore, the above implies that, conditioning on an agent u being a non-source agent, all the random bits used by u during the execution are accessible to all other agents.

- **Coordinated sources.** Even though non-source agents do not know who the sources are, we assume that sources do know who are the other sources. This means, in particular, that the sources can coordinate their actions.

Algorithm

Upon observation, each agent can alter its internal state (and in particular, its message to be seen by others) as well as its opinion. In reality, the updates of these variables may follow different constraints. In the case of ants for example, it may take a long time to change their message even if their internal state changes. As part our liberal approach, we allow agents to change any part of their internal state instantaneously.

The strategy in which agents update these variables is called "algorithm". As mentioned, algorithms can be randomized, that is, to determine the next action, agents can use the outcome of coin tosses in the sequence r (see the shared randomness assumption in Liberal assumptions). Overall, the action of an agent u at time t depends on:

1. the initial state of u in the charged initial configuration (including, in particular, the identity of u and whether or not it is a source),
2. the initial knowledge of u (including the system's parameters and the neutral configuration),
3. the time step t, and the list of its observations (history) up to time $t-1$, denoted $x_u^{(<t)}$,
4. the sequence of random bits r.

Convergence and time complexity

At any time, the opinion of an agent can be viewed as a binary *guess* function that is used to express its most knowledgeable guess of the correct opinion. The agents aim to minimize the probability that they fail to guess this opinion. In this context, it can be shown that the optimal guessing function is deterministic (see A remark about random guess functions, in the Appendix).

Definition 3. *We say that* convergence *has been achieved if one can specify a particular non-source agent v, for which it is guaranteed that its opinion is the correct opinion with probability at least $2/3$. The* time complexity *is the number of time steps (respectively, rounds) required to achieve convergence.*

We remark that the latter definition encompasses all three models considered.

Remark 1 (Different sampling rates of sources)**.** *We consider sources as agents in the population but remark that they can also be thought of as representing the environment. In this case, one may consider a different rate for sampling a source (environment) vs. sampling a typical agent. For example, the probability to observe any given source (or environment) may be x times more than the probability to observe any given non-source agent. This scenario can also be captured by a slight adaptation of our analysis. When x is an integer, we can alternatively obtain such a generalization by considering additional* artificial *sources in the system. Specifically, we replace each source u_i with a set of sources U_i consisting of x sources that coordinate their actions and behave identically (recall that we assume that sources know who are the other sources and can coordinate their actions), simulating the original behavior of u_i. Since the number of sources increases by a multiplicative factor of x, our lower bounds (see Theorem 4 and Corollary 14.1) decrease by a multiplicative factor of x^2.*

Related works in computer science

In *Rumor Spreading* problems (also referred to as *Broadcast*) a piece of information typically held by a single designated agent is to be disseminated to the rest of the population. It is the subject of a vast literature in theoretical computer science, and more specifically in the distributed computing community, see, *e.g.,* [3, 4, 6, 7, 8, 11, 13, 14, 16]. While some works assume a fixed topology, the canonical setting does not assume a network. Instead agents communicate through uniform $\mathcal{PUSH}/\mathcal{PULL}$ based interactions (including the *phone call* model), in which agents interact in pairs with other agents independently chosen at each time step uniformly at random from all agents in the population. The success of such protocols is largely due to their inherent simplicity and fault-tolerant resilience [10, 14]. In particular, it has been shown that under the $\mathcal{PUSH}$ model, there exists an efficient rumor spreading protocol that uses a single bit per message and can overcome flips in messages (noise) [11].

The line of research initiated by El-Gamal [9], also studies a broadcast problem with noisy interactions. The regime however is rather different from ours: all n agents hold a bit they wish to transmit to a single

receiver. This line of research culminated in the $\Omega(n \log\log n)$ lower bound on the number of messages shown in [13], matching the upper bound shown many years earlier in [12].

Several works have investigated algorithmic properties of networks with unstable topological structure, such as *ephemeral networks*, *evolving graphs* and *edge-Markovian evolving graphs* [2, 5, 1]. Such works prove analytical results assuming that the evolution of the topology satisfies certain constraints, and did not consider the case of noise affecting communication in conjunction with the dynamicity of the topology.

The lower bounds

Throughout this section we consider $\delta < 1/|\Sigma|$, such that $\frac{(1-\delta|\Sigma|)}{\delta s n} \leq \frac{1}{10}$. Our goal in this section is to prove the following result.

Theorem 4. *Assume that the relaxed δ-uniform noise criterion is satisfied.*

- *Let k be an integer. Any rumor spreading protocol on the* parallel-$\mathcal{PULL}(k)$ *model cannot converge in fewer rounds than*
$$\Omega\left(\frac{n\delta}{ks^2(1-\delta|\Sigma|)^2}\right).$$
- *Consider either the* sequential-$\mathcal{PULL}$ *or the* broadcast-$\mathcal{PULL}$ *model. Any rumor spreading protocol cannot converges in fewer time steps than*
$$\Omega\left(\frac{n^2\delta}{s^2(1-\delta|\Sigma|)^2}\right).$$

To prove the theorem, we first prove (in Reducing to the *broadcast-$\mathcal{PULL}$* Model) that an efficient rumor spreading algorithm in either the noisy *sequential-$\mathcal{PULL}$* model or the *parallel-$\mathcal{PULL}$*(k) model can be used to construct an efficient algorithm in the *broadcast-$\mathcal{PULL}$* model. The resulting algorithm has the same time complexity as the original one in the context of *sequential-$\mathcal{PULL}$* and adds a multiplicative factor of kn in the context of *parallel-$\mathcal{PULL}$*(k).

We then show how to relate the rumor spreading problem in *broadcast-$\mathcal{PULL}$* to a statistical inference test (Rumor Spreading and hypothesis testing). A lower bound on the latter setting is then achieved by adapting techniques from mathematical statistics (Proof of Theorem 7).

Remark 2. *The lower bound of Theorem 4 loses relevance when s is of order greater than $\sqrt{n}$. Indeed, the following simple protocol in the* broadcast-$\mathcal{PULL}$ *model turns out to match the lower bound. For simplicity's sake, let us consider the case of a binary alphabet $\Sigma = \{0,1\}$, and assume without loss of generality that the sources' opinion is 1. Each non-source agent, at each time step, chooses a random message u.a.r. in Σ, while each source agent always displays the correct message. After n time steps, the agents have collected n observations. If $s \gg \sqrt{1}0n$, a straightforward application of the Chernoff bound shows that with high probability at least $s/2$ of the n observations come from source agents. Thus, at most $n - s/2$ of the observations have distribution Bernoulli($\frac{1}{2}$), while at least $s/2$ of them are identically 1 before the effect of noise is taken into account. Since $s/2$ is of the same order of the standard deviation of the non-source messages, the agents have a good probability to correctly infer the correct opinion by choosing the most frequent message among the n observations.*

Reducing to the *broadcast-$\mathcal{PULL}$* Model

The following lemma establishes a formal relation between the convergence times of the models we consider. We assume all models are subject to the same noise distribution.

Lemma 5. *Any protocol operating in* sequential-$\mathcal{PULL}$ *can be simulated by a protocol operating in* broadcast-$\mathcal{PULL}$ *with the same time complexity. Moreover, for any integer $1 \leq k \leq n$, any protocol $\mathcal{P}$ operating in* parallel-$\mathcal{PULL}(k)$ *can be simulated by a protocol operating in* broadcast-$\mathcal{PULL}$ *with a time complexity that is kn times that of $\mathcal{P}$ in* parallel-$\mathcal{PULL}(k)$.

Proof. Let us first show how to simulate a time step of *sequential-$\mathcal{PULL}$* in the *broadcast-$\mathcal{PULL}$* model. Recall that in *broadcast-$\mathcal{PULL}$*, in each time step, all agents receive the same observation sampled u.a.r from the population. Upon drawing such an observation, all agents use their shared randomness to

generate a (shared) uniform random integer X between 1 and n. Then, the agent whose unique identity corresponds to X is the one processing the observation, while all other agents ignore it. This reduces the situation to a scenario in *sequential-$\mathcal{PULL}$*, and the agents can safely execute the original algorithm designed for that model.

As for simulating a time step of *parallel-$\mathcal{PULL}$*(k) in the *broadcast-$\mathcal{PULL}$* model, agents divide time steps in the latter model into *rounds*, each composing of precisely kn time steps. Recall that the model assumes that agents share clocks that start when the execution starts and tick at each time step. This implies that the agents can agree on the division of time into rounds, and can further agree on the round number. For an integer i, where $1 \leq i \leq kn$, during the i-th step of each round, only the agent whose identity is $(i \bmod n)+1$ receives the observation, while all other agents ignore it. Observe that receiving the observation doesn't imply that the agent processes this observation. In fact, it will store it in its memory until the round is completed, and process it only then. The aforementioned rule for receiving a message, ensures that when a round is completed in the *broadcast-$\mathcal{PULL}$* model, each agent receives precisely k independent uniform samples as it would in a round of *parallel-$\mathcal{PULL}$*(k). Therefore, at the end of each round $j \in \mathbb{N}^+$ in the *broadcast-$\mathcal{PULL}$* model, all agents can safely execute their actions in the j'th round of the original protocol designed for *parallel-$\mathcal{PULL}$*(k). This draws a precise bijection from rounds in *parallel-$\mathcal{PULL}$*(k) and rounds in *broadcast-$\mathcal{PULL}$*. The multiplicative overhead of kn simply follows from the fact that each round in *broadcast-$\mathcal{PULL}$* consists of kn time steps. □

Thanks to Lemma 5, Theorem 4 directly follows from the next theorem.

Theorem 6. *Consider the* broadcast-$\mathcal{PULL}$ *model and assume that the relaxed δ-uniform noise criterion is satisfied. Any rumor spreading protocol cannot converge in fewer time steps than*

$$\Omega\left(\frac{n^2\delta}{s^2(1-\delta|\Sigma|)^2}\right).$$

The remaining of the section is dedicated to proving Theorem 6. Towards achieving this, we view the task of guessing the correct opinion in the *broadcast-$\mathcal{PULL}$* model, given access to noisy samples, within the more general framework of distinguishing between two types of stochastic processes which obey some specific assumptions.

Rumor Spreading and hypothesis testing

Consider the following class of problems.

Adaptive Coin Distinguishing Task (`ACDT`). A *distinguisher* is presented with a sequence of observations taken from a coin of type η where $\eta \in \{0, 1\}$. We can think of the type η as initially set to 0 or 1 with probability 1/2 (independently of everything else). The goal of the distinguisher is to determine the type η, based on the observations.

More specifically, for a given time step t, denote the sequence of previous observations (up to, and including, time $t-1$) by

$$x^{(<t)} = (x^{(1)}, \ldots, x^{(t-1)}).$$

For each time t, we denote the probability distribution of the observation $X_\eta^{(t)} \in \Sigma$ that the distinguisher receives, given the type $\eta \in \{0, 1\}$ and the history of previous observations $x^{(<t)}$, as

$$P(X_\eta^{(t)} = m \mid x^{(<t)}). \tag{1}$$

We follow the common practice to use uppercase letters to denote random variables and lowercase letter to denote a particular realisation, e. g. $\mathbf{X}^{(\leq t)}$ for the sequence of observations up to time t, and $\mathbf{x}^{(\leq t)}$ for a corresponding realization.

Remark 3. *We note that* `ACDT` *generalizes in several respects the canonical problem of distinguishing between an unbiased coin and a coin with fixed bias ε (see,* e.g., *Chapter* 5 *in [17]). It is more general because 1. the probabilities of observations may vary adaptively as a function of the outcome of the previous samples, since the coins and $p_1^{(t)}(m)$ $P(X_0^{(t)} = m \mid x^{(<t)})$ and $P(X_1^{(t)} = m \mid x^{(<t)})$ in* (1) *actually depend on $x^{(<t)}$, the history of observations up to time $t-1$, and 2. instead of binary random variables we consider Σ-valued random variables.*

We next introduce, for each $m \in \Sigma$, the parameter

$$\varepsilon(m, x^{(<t)}) = P(X_1^{(t)} = m \mid x^{(<t)}) - P(X_0^{(t)} = m \mid x^{(<t)}).$$

Since, at all times t, it holds that $\sum_{m\in\Sigma} P(X_0^{(t)} = m \mid x^{(<t)}) = \sum_{m\in\Sigma} P(X_1^{(t)} = m \mid x^{(<t)}) = 1$, then $\sum_{m\in\Sigma} \varepsilon(m, x^{(<t)}) = 0$. We shall be interested in the quantity

$$d_\varepsilon(x^{(<t)}) := \sum_{m\in\Sigma} |\varepsilon(m, x^{(<t)})|,$$

which corresponds to the ℓ_1 distance between the distributions $P(X_0^{(t)} = m \mid x^{(<t)})$ and $P(X_1^{(t)} = m \mid x^{(<t)})$ given the sequence of previous observations.

The bounded family $\texttt{ACDT}(\varepsilon, \delta)$. We consider a family of instances of $\texttt{ACDT}$, called $\texttt{ACDT}(\varepsilon, \delta)$, governed by parameters ε and δ. Specifically, this family contains all instances of $\texttt{ACDT}$ such that for every t, and every history $x^{(<t)}$, we have:

- $d_\varepsilon(x^{(<t)}) \le \varepsilon$, and
- for every $m \in \Sigma$ such that $\varepsilon(m, x^{(<t)}) \neq 0$, we have $\delta \le P(X_\eta^{(t)} = m \mid x^{(<t)})$ for $\eta \in \{0, 1\}$.

In the rest of the current section, we show how Theorem 6, that deals with the *broadcast-$\mathcal{PULL}$* model, follows directly from the next theorem that concerns the adaptive coin distinguishing task, by setting

$$\varepsilon = \frac{2s(1 - \delta|\Sigma|)}{n}.$$

The actual proof of Theorem 7 appears in Proof of Theorem 7.

Theorem 7. *Consider any protocol for any instance of* $\texttt{ACDT}(\varepsilon, \delta)$*, The number of samples required to distinguish between a process of type* 0 *and a process of type* 1 *with probability of error less than* $\frac{1}{3}$ *is at least*

$$\frac{\ln 2}{9}\left(\frac{6(\delta - \varepsilon)^3}{\delta^3 - \delta^2\varepsilon + 3\delta\varepsilon^2 - \varepsilon^3}\right)\frac{\delta}{\varepsilon^2}.$$

In particular, if $10\varepsilon < \delta$*, then the number of necessary samples is* $\Omega\left(\frac{\delta}{\varepsilon^2}\right)$.

Proof of Theorem 6 assuming Theorem 7

Consider a rumor spreading protocol $\mathcal{P}$ in the *broadcast-$\mathcal{PULL}$* model. Fix a node u. We first show that running $\mathcal{P}$ by all agents, the perspective of node u corresponds to a specific instance of $\texttt{ACDT}(\frac{2s(1-\delta|\Sigma|)}{n}, \delta)$ called $\Pi(\mathcal{P}, u)$. We break down the proof of such correspondence into two claims.

The $\texttt{ACDT}$ instance $\Pi(\mathcal{P}, u)$. Recall that we assume that each agent knows the complete neutral initial configuration, the number of sources s, and the shared of random bits sequence r. We avoid writing such parameters as explicit arguments to $\Pi(\mathcal{P}, u)$ in order to simplify notation, however, we stress that what follows assumes that these parameters are fixed. The bounds we show hold for any fixed value of r and hence also when r is randomized.

Each agent is interested in discriminating between two families of charged initial configurations: Those in which the correct opinion is 0 and those in which it is 1 (each of these possibilities occurs with probability $\frac{1}{2}$). Recall that the correct opinion is determined in the 2nd stage of the charged initial configuration, and is independent form the choice of sources (1st stage).

We next consider the perspective of a generic non-source agent u, and define the instance $\Pi(\mathcal{P}, u)$ as follows. Given the history $x^{(<t)}$, we set $P(X_\eta^{(t)} = m \mid x^{(<t)})$, for $\eta \in \{0, 1\}$, to be equal to the probability that u observes message $m \in \Sigma$ at time step t of the execution $\mathcal{P}$. For clarity's sake, we remark that the latter probability is conditional on:

- the history of observations being $x^{(<t)}$,
- the sequence of random bits r,

- the correct opinion being $\eta \in \{0, 1\}$,
- the neutral initial configuration,
- the identity of u,
- the algorithm $\mathcal{P}$, and
- the system's parameters (including the distribution $f_{source-state}$ and the number of sources s).

Claim 8. *Let $\mathcal{P}$ be a correct protocol for the rumor spreading problem in* broadcast-$\mathcal{PULL}$ *and let u be an agent for which the protocol is guaranteed to produce the correct opinion with probability at least p by some time T (if one exists), for any fixed constant $p \in (0, 1)$. Then $\Pi(\mathcal{P}, u)$ can be solved in time T with correctness being guaranteed with probability at least p.*

Proof. Conditioning on $\eta \in \{0, 1\}$ and on the random seed r, the distribution of observations in the $\Pi(\mathcal{P}, u)$ instance follows precisely the distribution of observations as perceived from the perspective of u in *broadcast-$\mathcal{PULL}$*. Hence, if the protocol $\mathcal{P}$ at u terminates with output $j \in \{0, 1\}$ at round T, after the T-th observation in $\Pi(\mathcal{P}, u)$ we can set $\Pi(\mathcal{P}, u)$'s output to j as well. Given that the two stochastic processes have the same law, the correctness guarantees are the same. $\square$

Claim 9. $\Pi(\mathcal{P}, u) \in \mathtt{ACDT}\left(\frac{\mathtt{2(1-\delta|\Sigma|)s}}{\mathtt{n}}, \delta\right)$.

Proof. Since the noise in *broadcast-$\mathcal{PULL}$* flips each message $m \in \Sigma$ into any $m' \in \Sigma'$ with probability at least δ, regardless of the previous history and of $\eta \in \{0, 1\}$, at all times t we have

$$m \in \Sigma' \implies P(X_\eta^{(t)} = m \mid x^{(<t)}) \geq \delta.$$

Consider a message $m \in \Sigma \setminus \Sigma'$ (if such a message exists). By definition, such a message could only be received by observing a non-source agent. But given the same history $x^{(<t)}$, the same sequence of random bits r, and the same initial knowledge, the behavior of a non-source agent is the same, no matter what is the correct opinion η. Hence, for $m \in \Sigma \setminus \Sigma'$ we have $P(X_0^{(t)} = m \mid x^{(<t)}) = P(X_1^{(t)} = m \mid x^{(<t)})$, or in other words,

$$m \in \Sigma \setminus \Sigma' \implies \varepsilon(m, x^{(<t)}) = 0.$$

It remains to show that $d_\varepsilon(x^{(<t)}) \leq \frac{2(1-\delta|\Sigma|)s}{n}$. Let us consider two executions of the rumor spreading protocol, with the same neutral initial configuration, same shared sequence of random bits r, same set of sources, except that in the first the correct opinion is 0 while in the other it is 1. Let us condition on the history of observations $x^{(<t)}$ being the same in both processes.

As mentioned, given the same history $x^{(<t)}$, the behavior of a non-source agent is the same, regardless of the correct opinion η. It follows that the difference in the probability of observing any given message is only due to the event that a source is observed. Recall that the number of sources is s. Therefore, the probability of observing a source is s/n, and we may write as a first approximation $\varepsilon(m, x^{(<t)}) \leq s/n$. However, we can be more precise. In fact, $\varepsilon(m, x^{(<t)})$ is slightly smaller than s/n, because the noise can still affect the message of a source.

We may interpret $\varepsilon(m, x^{(<t)})$ as the following difference. For a source $v \in S$, let m_η^v be the message of u assuming the given history $x^{(<t)}$ and that v is of type $\eta \in \{0, 1\}$ (the message m_η^v is deterministically determined given the sequence r of random bits, the neutral initial configuration, the parameters of the system, and the identity of v). Let $\alpha_{m',m}$ be the probability that the noise transforms a message m' into a message m. Then

$$\varepsilon(m, x^{(<t)}) = \frac{1}{n} \sum_{v \in S} (\alpha_{m_1^v, m} - \alpha_{m_0^v, m}),$$

and

$$d_\varepsilon(x^{(<t)}) = \sum_{m \in \Sigma} |\varepsilon(m, x^{(<t)})| \leq \frac{1}{n} \sum_{m \in \Sigma} \sum_{v \in S} |\alpha_{m_1^v, m} - \alpha_{m_0^v, m}|. \tag{2}$$

By the definition of $\mathtt{ACDT}(\varepsilon, \delta)$, it follows that either $\alpha_{m_1^v, m} = \alpha_{m_0^v, m}$ (if $\varepsilon(m, x^{(<t)}) = 0$) or $\delta \leq \alpha_{m_1^v, m}, \alpha_{m_0^v, m} \leq 1 - \delta$ (if $\varepsilon(m, x^{(<t)}) \neq 0$). Thus, to bound the right hand side in (2), we can use the following claim (proven in Proof of Claim 10)

Claim 10. *Let P and Q be two distributions over a universe Σ such that for any element $m \in \Sigma$, $\delta \leq P(m), Q(m) \leq 1-\delta$. Then $\sum_{m\in\Sigma}|P(m)-Q(m)| \leq 2(1-\delta|\Sigma|)$.*

Applying Claim 10 for a fixed $v \in S$ to distributions $(\alpha_{m_0^v,m})_m$ and $(\alpha_{m_1^v,m})_m$, we obtain

$$\frac{1}{n}\sum_{m\in\Sigma}\sum_{v\in S}|\alpha_{m_1^v,m}-\alpha_{m_0^v,m}| \leq \frac{1}{n}2\sum_{v\in S}(1-\delta|\Sigma|) \leq \frac{2(1-\delta|\Sigma|)s}{n}.$$

Hence, we have $\Pi(\mathcal{P}) \in \mathtt{ACDT}\left(\frac{2(1-\delta|\Sigma|)\mathtt{s}}{\mathtt{n}}, \delta\right)$, establishing Claim 9. $\square$

Thanks to Claims 8 and 9, Theorem 6 regarding the *broadcast-$\mathcal{PULL}$* model becomes a direct consequence of Theorem 7 on the adaptive coin distinguishing task, taking

$$\varepsilon = \frac{2(1-\delta|\Sigma|)s}{n}\ .$$

More precisely, the assumption $\frac{(1-\delta|\Sigma|)}{\delta sn} \leq c$ for some small constant c, ensures that $\frac{\varepsilon}{\delta} \leq c$ as required by Theorem 7. The lower bound $\Omega\left(\frac{\varepsilon^2}{\delta}\right)$ corresponds to

$$\Omega\left(\frac{n^2\delta}{(1-\delta|\Sigma|)^2s^2}\right)\ .$$

This concludes the proof of Theorem 6. To establish our results it remains to prove Theorem 7.

Proof of Theorem 7

We start by recalling some facts from Hypothesis Testing.

We use the notation $\log(\cdot)$ to denote the base 2 logarithms, i.e., $\log_2(\cdot)$ and for a probability distribution P, use the notation $P(x)$ as a short for $P(X=x)$. First let us recall two standard notions of (pseudo) distances between probability distributions. Given two discrete distributions P_0, P_1 over a probability space Ω with the same support, the *total variation distance* is defined as

$$TV(P_0,P_1) := \frac{1}{2}\sum_{x\in\Omega}|P_0(x)-P_1(x)|,$$

and the Kullback-Leibler divergence $KL(P_0,P_1)$ is defined as

$$KL(P_0,P_1) := \sum_{x\in\Omega}P_0(x)\log\frac{P_1(x)}{P_0(x)}.$$

The assumption that the support is the same is not necessary but it is sufficient for our purposes, and is thus made for simplicity's sake.

The following lemma shows that, when trying to discriminate between distributions P_0, P_1, the total variation relates to the smallest error probability we can hope for.

Lemma 11 (Neyman-Pearson [17, Lemma 5.3 and Proposition 5.4])**.** *Let P_0, P_1 be two distributions. Let $X \in \Omega$ be a random variable of distribution either P_0 or P_1. Consider a (possibly probabilistic) mapping $f:\Omega\to\{0,1\}$ that attempts to "guess" whether the observation X was drawn from P_0 (in which case it outputs 0) or from P_1 (in which case it outputs 1). Then, we have the following lower bound,*

$$P_0\left(f(X)=1\right)+P_1\left(f(X)=0\right) \geq 1-TV(P_0,P_1).$$

The total variation is related to the KL divergence by the following inequality.

Lemma 12 (Pinsker [17, Lemma 5.8])**.** *For any two distributions P_0, P_1,*

$$TV(P_0,P_1) \leq \sqrt{KL\left(P_0,P_1\right)}.$$

We are now ready to prove the theorem.

Proof of Theorem 7. Let us define $P_\eta\left(\,\cdot\,\right) = P\left(\,\cdot \mid \text{"correct distribution is } \eta\text{"}\right)$ for $\eta \in \{0,1\}$. We denote $P_\eta^{(\le t)}$, $\eta \in \{0,1\}$, the two possible distributions of $\mathbf{X}^{(\le t)}$. We refer to $P_0^{(\le t)}$ as the distribution of *type* 0 and to $P_1^{(\le t)}$ as the distribution of *type* 1. Furthermore, we define the *correct type* of a sequence of observations $\mathbf{X}^{(\le t)}$ to be 0 if the observations are sampled from $P_0^{(\le t)}$, and to be 1 if they are sampled from $P_1^{(\le t)}$.

After t observations $\mathbf{x}^{(\le t)} = (x^{(1)}, \dots, x^{(t)})$ we have to decide whether the distribution is of type 0 or 1. Our goal is to maximize the probability of guessing the type of the distribution, observing $\mathbf{X}^{(\le t)}$, which means that we want to minimize

$$P\left(f(\mathbf{X}^{(\le t)}) \neq \text{"correct type"}\right) = \sum_{\eta\in\{0,1\}} P_\eta\left(f(\mathbf{X}^{(\le t)}) = 1-\eta\right) P\left(\text{"correct type is } \eta\text{"}\right). \tag{3}$$

Recall that the correct type is either 0 or 1 with probability $\frac{1}{2}$. Thus, the error probability described in (3) becomes

$$\frac{1}{2}P_0\left(f(\mathbf{X}^{(\le t)}) = 1\right) + \frac{1}{2}P_1\left(f(\mathbf{X}^{(\le t)}) = 0\right). \tag{4}$$

By combining Lemmas 11 and 12 with $X = \mathbf{X}^{(\le t)}$ and $P_\eta = P_\eta^{(\le t)}$ for $\eta = 0,1$, we get the following Theorem. Although for convenience we think of f as a deterministic function, it could in principle be randomized (see A remark about random guess functions, in the Appendix).

Theorem 13. *Let f be any guess function. Then*

$$P_0\left(f(\mathbf{X}^{(\le t)}) = 1\right) + P_1\left(f(\mathbf{X}^{(\le t)}) = 0\right) \ge 1 - \sqrt{KL\left(P_0^{(\le t)}, P_1^{(\le t)}\right)}.$$

Theorem 13 implies that for the probability of error to be small, it must be the case that the term $KL\left(P_0^{(\le t)}, P_1^{(\le t)}\right)$ is large. Our next goal is therefore to show that in order to make this term large, t must be large.

Note that $P_\eta^{(\le T)}$ for $\eta \in \{0,1\}$ cannot be written as the mere product of the marginal distributions of the $X^{(t)}$s, since the observations at different times may not necessarily be independent. Nevertheless, we can still express the term $KL(P_0^{(\le T)}, P_1^{(\le T)})$ as a sum, using the Chain Rule for KL divergence. It yields

$$KL(P_0^{(\le T)}, P_1^{(\le T)}) = \sum_{t\le T} KL(P_0(x^{(t)} \mid x^{(<t)}), P_1(x^{(t)} \mid x^{(<t)})), \tag{5}$$

where

$$\begin{aligned} &KL(P_0(x^{(t)} \mid x^{(<t)}), P_1(x^{(t)} \mid x^{(<t)})) \\ &\qquad := \sum_{x^{(<t)}\in\Sigma^{t-1}} P_0(x^{(<t)}) \sum_{x^{(t)}\in\Sigma} P_0(x^{(t)} \mid x^{(<t)}) \log \frac{P_0(x^{(t)} \mid x^{(<t)})}{P_1(x^{(t)} \mid x^{(<t)})}. \\ &\qquad = \sum_{x^{(<t)}\in\Sigma^{t-1}} P_0(x^{(<t)}) \sum_{m\in\Sigma} P_0(X_0^{(t)} = m \mid x^{(<t)}) \log \frac{P(X_0^{(t)} = m \mid x^{(<t)})}{P(X_1^{(t)} = m \mid x^{(<t)})}. \end{aligned} \tag{6}$$

Since we are considering an instance of $\texttt{ACDT}\,(\varepsilon,\delta)$, we have

- $d_\varepsilon(x^{(<t)}) = \sum_{m\in\Sigma} |\varepsilon(m, x^{(<t)})| \le \varepsilon$, and
- for every $m \in \Sigma$ such that $\varepsilon(m, x^{(<t)}) \neq 0$, it holds that $\delta \le P_\eta(X_0^{(t)} = m \mid x^{(<t)})$ for $\eta \in \{0,1\}$.

We make use of the previous facts to upper bound the KL divergence terms in the right hand side of (6), as follows (recall that we omit the dependency of $p^{(<t)}$ and $\varepsilon^{(<t)}$ on the past observations $x^{(<T)} \in \Sigma^{t-1}$, in the interest of readability).

$$\begin{aligned} &KL(P_0(x^{(t)} \mid x^{(<t)}), P_1(x^{(t)} \mid x^{(<t)})) \\ &= \sum_{x^{(<t)}\in\Sigma^{t-1}} P_0(x^{(<t)}) \sum_{m\in\Sigma} \left(P(X_0^{(t)} = m \mid x^{(<t)}) \log \frac{P(X_0^{(t)} = m \mid x^{(<t)})}{P(X_0^{(t)} = m \mid x^{(<t)}) + \varepsilon(m, x^{(<t)})} \right) \\ &= -\sum_{x^{(<t)}} P_0(x^{(<t)}) \sum_{m\in\Sigma} \left(P(X_0^{(t)} = m \mid x^{(<t)}) \log \left(1 + \frac{\varepsilon(m, x^{(<t)})}{P(X_0^{(t)} = m \mid x^{(<t)})} \right) \right). \end{aligned} \tag{7}$$

Recall that we assume

$$\frac{\varepsilon(m,x^{(<t)})}{P(X_0^{(t)}=m\mid x^{(<t)})}\le\frac{\varepsilon(m,x^{(<t)})}{\delta}\le\frac{\varepsilon}{\delta}.$$

We make use of the following claim, which follows from the Taylor expansion of $\log(1+u)$ around 0. More details can be found in the Appendix, Proof of Claim 14.

Claim 14. *Let $x\in[-a,a]$ for some $a\in(0,1)$. Then* $|\log(1+x)-x+x^2/2|\le\frac{x^3}{3(1-a)^3}$.

Using Claim 14 with $a=\frac{\varepsilon}{\delta}$, we can bound the inner sum appearing in (7) from above and below with

$$\frac{1}{\ln 2}\sum_{m\in\Sigma}\left(\varepsilon(m,x^{(<t)})-\frac{1}{2}\frac{(\varepsilon(m,x^{(<t)}))^2}{P(X_0^{(t)}=m\mid x^{(<t)})}\pm\frac{\delta^3}{3(\delta-\varepsilon)^3}\left(\frac{(\varepsilon(m,x^{(<t)}))^3}{P(X_0^{(t)}=m\mid x^{(<t)})^2}\right)\right).\tag{8}$$

Since $\sum_m|\varepsilon(m,x^{(<t)})|\le\varepsilon$, we also have that $\sum_m(\varepsilon(m,x^{(<t)}))^2\le\varepsilon^2$. The latter bound, together with the fact that $P(X_0^{(t)}=\tilde m\mid x^{(<t)})\ge\delta$ for any $\tilde m\in\Sigma$ such that $\varepsilon(\tilde m,x^{(<t)})\neq 0$, implies

$$\sum_m\frac{(\varepsilon(m,x^{(<t)}))^2}{P(X_0^{(t)}=m\mid x^{(<t)})}\le\frac{\varepsilon^2}{\delta}\ .\tag{9}$$

Finally, we can similarly bound the term $\sum_{m\in\Sigma}\left((\varepsilon(m,x^{(<t)}))^3/P(X_0^{(t)}=m\mid x^{(<t)})^2\right)$ with

$$\sum_{m\in\Sigma}\left((\varepsilon(m,x^{(<t)}))^3/P(X_0^{(t)}=m\mid x^{(<t)})^2\right)\le\frac{\varepsilon^3}{\delta^2}.\tag{10}$$

Recall that $\sum_m\varepsilon(m,x^{(<t)})=0$, thus the first term in (8) disappears. Hence, substituting the bounds (9) and (10) in (8), we have

$$\begin{aligned}\sum_m P(X(t)=m|x(<t))&\left|\log\left(1+\frac{\varepsilon(m,x^{(<t)})}{P(X_0^{(t)}=m\mid x^{(<t)})}\right)\right|\\&\le\frac{1}{\ln 2}\left(\frac{1}{2}\frac{\varepsilon^2}{\delta}+\frac{\delta\varepsilon^3}{3(\delta-\varepsilon)^3}\right)\cdot\sum_m P(X(t)=m|x(<t))\\&\le\frac{1}{\ln 2}\left(\frac{1}{2}+\frac{\delta^2\varepsilon}{3(\delta-\varepsilon)^3}\right)\frac{\varepsilon^2}{\delta}\cdot 1.\end{aligned}\tag{11}$$

If we define the right hand side (11) to be $W(\varepsilon,\delta)$ and we substitute the previous bound in (7), we get

$$KL(P_0(x^{(t)}\mid x^{(<t)}),P_1(x^{(t)}\mid x^{(<t)}))\le W(\varepsilon,\delta),$$

and combining the previous bound with (5), we can finally conclude that for any integer T, we have

$$KL(P_0^{(\le T)},P_1^{(\le T)})\le T\cdot W(\varepsilon,\delta).$$

Thus, from Theorem 13 and the latter bound, it follows that the error under a uniform prior of the source type, as defined in (4), is at least

$$\frac{1}{2}P_0\left(f(\mathbf{X}^{(\le t)})=1\right)+\frac{1}{2}P_1\left(f(\mathbf{X}^{(\le t)})=0\right)\ge\frac{1}{2}-\frac{1}{2}\sqrt{KL(P_0^{(\le T)},P_1^{(\le T)})}\ge\frac{1}{2}-\frac{1}{2}\sqrt{T\cdot W(\varepsilon,\delta)}.$$

Hence, the number of samples T needs to be greater than

$$\frac{1}{9}\frac{1}{W(\varepsilon,\delta)}=\frac{\ln 2}{9}\left(\frac{6(\delta-\varepsilon)^3}{\delta^3-\delta^2\varepsilon+3\delta\varepsilon^2-\varepsilon^3}\right)\frac{\delta}{\varepsilon^2}.$$

to allow the possibility that the error be less than 1/3.

In particular, if we assume that $10\varepsilon<\delta$, then we can bound

$$\frac{\delta^2\varepsilon}{3(\delta-\varepsilon)^3}\le\frac{\delta^3}{10}\cdot\frac{1}{3(9/10)^3\delta^3}\le\frac{100}{2187}.$$

It follows that (11) can be bounded with

$$W(\varepsilon,\delta) \leq \frac{\delta}{\varepsilon^2}\frac{1}{\ln 2}\left(\frac{1}{2}+\frac{100}{2187}\right) \leq 0.79\,,$$

and so

$$\frac{1}{9}\frac{1}{W(\varepsilon,\delta)} \geq 0.14\cdot\frac{\delta}{\varepsilon^2} = \Omega\left(\frac{\delta}{\varepsilon^2}\right).$$

This completes the proof of Theorem 7 and hence of Theorem 6. □

Detectable sources

In this section, we aim to prove the following.

Corollary 14.1. *Consider the setting in which sources are reliably detectable. Assume that the relaxed δ-uniform noise criterion is satisfied and let $T = \left(\frac{n^2\delta}{s^2(1-\delta|\Sigma|)^2}\right)^{1/3}$.*

- *Consider the* sequential-$\mathcal{PULL}$ *model. Assume that $sT \geq C\log n$, for a large enough constant C. Any rumor spreading scheme cannot converge in less than $\Omega(T)$ time steps.*
- *Let k be an integer. Assume that $sT/k \geq C\log n$, for a large enough constant C. Any rumor spreading protocol in the* parallel-$\mathcal{PULL}(k)$ *model cannot converge in less than $\Omega(T/k)$ rounds.*

Proof. Let us start with the first item of the corollary, namely the lower bound in the *sequential-$\mathcal{PULL}$* model. For any step t, let $S(t)$ denote the set of sources together with the agents that have directly observed at least one of the sources at some point up to time t. We have $S = S(0) \subseteq S(1) \subseteq S(2) \subseteq \ldots$. The size of the set $S(t)$ is a random variable which is expected to grow at a moderate speed. Specifically, letting $s' = \frac{11}{10}\cdot s\cdot T$, we obtain:

Claim 15. *With probability at least $1-n^{-10}$, we have $|S(T)| \leq s'$.*

Proof of Claim 15. The variable $S(T)$ may be written as a sum of indicator variables

$$\begin{aligned} S(T) &= \sum_{i=1}^{n} \mathbb{1}(\text{Agent } i \text{ observed at least one source before step } t) \\ &\leq \sum_{i=1}^{n}\sum_{r\leq T} \mathbb{1}(\text{Agent } i \text{ observes a source on step } r). \end{aligned}$$

This last expression is a sum of $n\cdot T$ independent Bernoulli variables with parameter s/n. In other terms, it is a binomial variable with probability s/n and $T{\cdot}n$ trials. By a standard Chernoff bound the probability that it deviates by a multiplicative factor $\frac{11}{10}$ from its mean $s\cdot T$ is less than $\exp(-\Omega(sT)) \leq n^{-10}$. The last bound holds because we assume $sT \geq C\log n$ for some large enough constant C. □

Denote by $\mathcal{E}$ the event that $|S(t)| \leq s'$ for every $t \leq T$. Using Claim 15, we know that $P(\mathcal{E}) \geq 1-n^{-10}$. Our next goal is to prove that the probability ρ that a given agent correctly guesses the correct opinion is low for any given time $t \leq cT$, where c is a small constant. For this purpose, we condition on the highly likely event $\mathcal{E}$. Removing this conditioning will amount to adding a negligible term (of order at most n^{-10}) to ρ.

In order to bound ρ, we would like to invoke Theorem 6 with the number of sources upper bounded by s'. Let us explain why it applies in this context. To begin with, we may adversarially assume (from the perspective of the lower bound) that all agents in $S(t)$ learn the value of the correct bit to spread. Thus, they essentially become "sources" themselves. In this case the number of sources varies with time, but the proof of Theorem 6 can easily be shown to cover this case as long as s (i.e., s' here) is an upper bound on the number of sources at all times. We can therefore safely apply Theorem 6 with s'. By the choice of T,

$$T = \Theta\left(\frac{n^2\delta}{(s')^2(1-\delta|\Sigma|)^2}\right).$$

Hence, we can set c to be a sufficiently small constant such that for all times $t \leq cT$, the probability of guessing correctly, even in this adversarial scenario, is less than 1/3. In other words, we have $\rho \leq 1/3$. All together, this yields a lower bound of $\Omega(T)$ on the convergence time.

As for the *parallel-$\mathcal{PULL}$*(k) model, the argument is similar. After $T' = T/k$ parallel rounds, using a similar claim as Claim 15, we have that with high probability, at most $\mathcal{O}(ksT')$ agents have directly observed one of the s sources by time T'. Applying Theorem 6 with $s'' = \mathcal{O}(ksT') = \mathcal{O}(sT)$ yields a lower bound (in terms of samples in the broadcast model) of

$$\Theta\left(\frac{n^2\delta}{(s'')^2(1-\delta|\Sigma|)^2}\right) = \Theta\left(\frac{n^2\delta}{s^2T^2(1-\delta|\Sigma|)^2}\right) = \Theta(T).$$

The last line follows by choice of T. Hence T is a lower bound on the number of samples, which is attained in T' rounds of *parallel-$\mathcal{PULL}$*(k) model. □

References

[1] E. C. Akrida, L. Gasieniec, G. B. Mertzios, and P. G. Spirakis. Ephemeral networks with random availability of links: The case of fast networks. *Journal of Parallel and Distributed Computing*, 87:109–120, January 2016.

[2] C. Avin, M. Kouck, and Z. Lotker. How to Explore a Fast-Changing World (Cover Time of a Simple Random Walk on Evolving Graphs). In *ICALP*, number 5125 in LNCS, pages 121–132. 2008.

[3] L. Boczkowski, A. Korman, and E. Natale. Minimizing message size in stochastic communication patterns: Fast self-stabilizing protocols with 3 bits. In *SODA*, pages 2540–2559, 2017.

[4] K. Censor-Hillel, B. Haeupler, J. A. Kelner, and P. Maymounkov. Global computation in a poorly connected world: fast rumor spreading with no dependence on conductance. In *STOC*, pages 961–970, 2012.

[5] A. Clementi, A. Monti, F. Pasquale, and R. Silvestri. Information Spreading in Stationary Markovian Evolving Graphs. *IEEE Transactions on Parallel and Distributed Systems*, 22(9):1425–1432, September 2011.

[6] A. Demers, D. Greene, C. Hauser, W. Irish, J. Larson, S. Shenker, H. Sturgis, D. Swinehart, and D. Terry. Epidemic algorithms for replicated database maintenance. In *PODC*, 1987.

[7] B. Doerr and M. Fouz. Asymptotically optimal randomized rumor spreading. *Electronic Notes in Discrete Mathematics*, 38:297–302, 2011.

[8] B. Doerr, L. A. Goldberg, L. Minder, T. Sauerwald, and C. Scheideler. Stabilizing consensus with the power of two choices. In *SPAA*, pages 149–158, 2011.

[9] A. El-Gamal. Open problems presented at the 1984 workshop on specific problems in communication and computation sponsored by bell communication research.

[10] R. Elsässer and T. Sauerwald. On the runtime and robustness of randomized broadcasting. *Theor. Comput. Sci.*, 410(36):3414–3427, 2009.

[11] O. Feinerman, B. Haeupler, and A. Korman. Breathe before speaking: efficient information dissemination despite noisy, limited and anonymous communication. In *PODC, 2014*.

[12] R. G. Gallager. Finding parity in a simple broadcast network. *IEEE Trans. Inf. Theor.*, 34(2):176–180, 2006.

[13] N. Goyal, G. Kindler, and M. E. Saks. Lower bounds for the noisy broadcast problem. *SIAM Journal of Computing*, 37(6):1806–1841, 2008.

[14] R. M. Karp, C. Schindelhauer, S. Shenker, and B. Vöcking. Randomized rumor spreading. In *FOCS*, pages 565–574, 2000.

[15] A. Korman, E. Greenwald, and O. Feinerman. Confidence sharing: An economic strategy for efficient information flows in animal groups. *PLoS Computational Biology*, 10(10), 2014.

[16] B. Pittel. On spreading a rumor. *SIAM Journal of Applied Mathematics*, 47(1):213–223, 1987.

[17] P. Rigollet. High dimensional statistics. *Lecture notes for course 18S997.*, 2015.

Missing proofs

Proof of Claim 10

The proof of the Claim could be slightly shortened using the well-known fact that the l_1 distance between two probability distribution equals two times the total variation between them. We derive a proof from first principles for completeness.

In general, define Σ_+, Σ_- and $\Sigma_=$ respectively as $\{m : P(m) > Q(m)\}$, $\{m : P(m) < Q(m)\}$ and $\{m : P(m) = Q(m)\}$. We have

$$\begin{aligned}\sum_{m\in\Sigma} |P(m) - Q(m)| &= \sum_{m\in\Sigma_+} (P(m) - Q(m)) + \sum_{m\in\Sigma_-} (Q(m) - P(m)) \\ &= P(\Sigma_+) - Q(\Sigma_+) + Q(\Sigma_-) - P(\Sigma_-) \\ &= 1 - P(\Sigma_-) - P(\Sigma_=) - Q(\Sigma_+) + 1 - Q(\Sigma_+) - Q(\Sigma_=) - P(\Sigma_-) \\ &= 2 - 2P(\Sigma_-) - 2Q(\Sigma_+) - P(\Sigma_=) - Q(\Sigma_=).\end{aligned}$$

Note that for any set S, $Q(S) \geq \delta|S|$ using the fact that $Q(m) \geq \delta$ for any m and similarly for P. Thus the last line is bounded by $2 - 2\delta(|\Sigma_=| + |\Sigma_-| + |\Sigma_=|) = 2(1 - \delta|\Sigma|)$.

This completes the proof of Claim 10.

□

A remark about random guess functions

In this section, we show that we may relax the guessing function f to be probabilistic. In other words, allowing $P(f(\tilde{\mathbf{x}}^{(\leq t)}) = 1) = p$ for some $\tilde{\mathbf{x}}^{(\leq t)}$ and some probability $0 < p < 1$, would not allow to reduce further the value of (4). Indeed, if we consider a probabilistic $f(\tilde{\mathbf{x}}^{(\leq t)})$ in (4), we could rewrite the latter as a convex combination of deterministic guessing functions. To illustrate this, consider for example the case in which f is random only on a particular input $\tilde{\mathbf{x}}^{(\leq t)}$, and define the two deterministic guessing function

$$f_1(\mathbf{x}^{(\leq t)}) = \begin{cases} 1 & \text{if } \mathbf{x}^{(\leq t)} = \tilde{\mathbf{x}}^{(\leq t)}, \\ f(\mathbf{x}^{(\leq t)}) & \text{otherwise,} \end{cases} \quad \text{and} \quad f_0(\mathbf{x}^{(\leq t)}) = \begin{cases} 0 & \text{if } \mathbf{x}^{(\leq t)} = \tilde{\mathbf{x}}^{(\leq t)}, \\ f(\mathbf{x}^{(\leq t)}) & \text{otherwise.} \end{cases}$$

From the above definition (and the law of total probability), it follows that

$$P_\eta\left(f(\mathbf{X}^{(\leq t)}) = 1\right) = pP_\eta\left(f_1(\mathbf{X}^{(\leq t)}) = 1\right) + (1-p)P_\eta\left(f_0(\mathbf{X}^{(\leq t)}) = 1\right) \qquad \text{for } \eta \in \{0, 1\},$$

which means

$$\begin{aligned}&\frac{1}{2}P_0\left(f(\mathbf{X}^{(\leq t)}) = 1\right) + \frac{1}{2}P_1\left(f(\mathbf{X}^{(\leq t)}) = 0\right) \\ &= \frac{1}{2}\left(pP_0\left(f_1(\mathbf{X}^{(\leq t)}) = 1\right) + (1-p)P_0\left(f_0(\mathbf{X}^{(\leq t)}) = 1\right)\right) \\ &\quad + \frac{1}{2}\left(pP_1\left(f_1(\mathbf{X}^{(\leq t)}) = 0\right) + (1-p)P_1\left(f_0(\mathbf{X}^{(\leq t)}) = 0\right)\right).\end{aligned}$$

The above calculation can be generalized to the case in which f is random on any subset of inputs (possibly all). Thus, our results still hold for probabilistic guess functions. Informally, this means that we can allow agents to take decisions by “flipping a coin”. We assume f to be deterministic for the sole purpose of easing the presentation.

Proof of Claim 14

Using a Taylor expansion of $\log(1+u)$ of order 3 around 0 and the Remainder Theorem, we obtain, for any $x \in [-a, a]$,

$$|\log(1+x) - x + x^2/2| \leq \frac{x^3}{6} \max_{y\in[-a,a]} (\log(1+y))^{(3)},$$

where $f^{(3)}$ for a function f stands for the third derivative of f. Since

$$(\log(1+y))^{(3)} = \frac{2}{(1+y)^3},$$

Claim 14 follows. □
